# Integrating a Group-Based, Early Childhood Parenting Intervention Into Primary Health Care Services in Rural Bangladesh: A Cluster-Randomized Controlled Trial

**DOI:** 10.3389/fped.2022.886542

**Published:** 2022-06-10

**Authors:** Syeda Fardina Mehrin, Mohammed Imrul Hasan, Fahmida Tofail, Shamima Shiraji, Deborah Ridout, Sally Grantham-McGregor, Jena D. Hamadani, Helen Baker-Henningham

**Affiliations:** ^1^International Centre for Diarrhoeal Disease Research, Bangladesh, Dhaka, Bangladesh; ^2^UCL Great Ormond Street Institute of Child Health, London, United Kingdom; ^3^School of Human and Behavioural Sciences, Bangor University, Bangor, United Kingdom

**Keywords:** parenting, child development, malnutrition, integrating into government services, primary health care, low- and middle-income countries

## Abstract

**Background:**

Over 250 million children globally do not reach their developmental potential. We tested whether integrating a group-based, early childhood parenting program into government healthcare clinics improved children’s development, growth, and behavior.

**Methods:**

We conducted a cluster-randomized controlled trial in 40 community clinics in the Kishorganj district of Bangladesh. We randomly assigned clinics (1:1) to deliver a group-based parenting interventions or to a comparison group that received no intervention. Participants were children aged 5–24 months, with weight-for-age *z*-score of ≤ −1.5 SDs of the WHO standards, living within a thirty-minute walking distance from the clinic (*n* = 419 intervention, 366 control). Government health staff facilitated parenting sessions in the clinic with groups of four mother/child dyads fortnightly for one year as part of their routine duties. Primary outcomes measured at baseline and endline were child development assessed using the Bayley scales, child behaviors during the test by tester ratings, and child growth. The trial is registered at ClinicalTrials.gov, NCT02208531.

**Findings:**

91% of children were tested at endline (396 intervention, 319 control). Multilevel analyses showed significant benefits of intervention to child cognition (effect size 0.85 SDs, 95% CI: 0.59, 1.11), language (0.69 SDs, 0.43, 0.94), and motor development (0.52 SDs, 0.31, 0.73), and to child behaviors during the test (ranging from 0.36 SDs, 0.14, 0.58, to 0.53 SDs, 0.35, 0.71). There were no significant effects on growth.

**Conclusion:**

A scalable parenting intervention, integrated into existing government health services and implemented by government health staff, led to significant benefits to child development and behavior.

## Introduction

Poor development in disadvantaged children under 5 years of age is a major problem in low- and middle-income countries (LMIC), leading to lifelong functional and economic consequences (1). There is strong evidence that early childhood development (ECD) parenting interventions, focusing on psychosocial stimulation, benefit these children’s development (2). To extend the reach of ECD parenting interventions, we need information on the best methods of implementing ECD programmes at scale. Many experts recommend integrating into the health services (3). The goal is for health staff to run ECD interventions as well as their routine tasks, which is potentially cost-effective, but there are few evaluations of this approach (4, 5). Moreover, researchers have often funded the health workers (6, 7).

In Bangladesh, children living in poverty show a rapid decline in cognitive and language development from 7-months through to 5-years of age (8). We have previously evaluated an ECD home-visiting, parenting program in Bangladesh (adapted from the Jamaican home-visiting program, now called Reach-Up) and conducted four randomized controlled trials in which locally-hired women conducted weekly parenting sessions with mother/child dyads at home or in a clinic setting (9–12). The benefits to child development from this approach were small-to-moderate (ES = 0.21–0.38 SD). However, individual sessions are costly and it is difficult to reach large numbers of disadvantaged children.

We developed an ECD parenting intervention that could be integrated into the primary health care clinics and thus facilitate scaling-up ECD interventions for at-risk children and we conducted a trial using the health care workers (HCW) to deliver the parenting sessions as part of their usual tasks. In addition, instead of mother/child dyads attending individual sessions every week, they attended in pairs every 2 weeks. Surprisingly, the children showed much larger benefits to cognition and language development (1.1–1.3 SD) than previously found in Bangladesh (13). Given the exceptionally large benefits to child outcomes compared with individual home-visiting interventions, or most other parenting interventions elsewhere (2), it was important to investigate the robustness of the findings (14, 15). We decided to replicate the intervention in another trial, but with several modifications to make it more suitable for wide scale dissemination. Firstly, groups of four mothers and children attended the session, potentially doubling the coverage per HCW. Groups of four were the largest number that could be accommodated inside the clinics due to space constraints. Secondly, we adapted the Reach-Up intervention to make it suitable for use with a wider age-range of children at each session by presenting play activities in six- or twelve-month age bands rather than into monthly age bands used in the pair curriculum. Thirdly, we reduced the variety of play materials used in the intervention by half, with children participating in one toy activity per session rather than two. Full details of the process of adaptation have been published previously (16).

In this study, we evaluated the effects of the ECD group-based parenting program on child cognition, language and motor development, behavior, and nutritional status.

## Methods

### Study Design and Participants

In Bangladesh, there are more than 13,000 community clinics that deliver primary health care across the country. We conducted a two-arm, single-blind, cluster randomized trial with parallel assignment in forty clinics in the rural Kishorganj district of Bangladesh, located approximately 100 km from Dhaka city. Clinic was the unit of randomization to reduce contamination between the groups as the intervention was integrated into clinic services and was implemented by existing clinic staff. We selected two rural subdistricts in Kishorganj with a total of seventy-four community clinics. An independent statistician randomly selected twenty clinics from each subdistrict (*n* = 40 clinics) to participate in this study. No clinics refused to participate.

Inclusion criteria for children were: weight for age (WAZ) ≤ −1.5 SD, singleton birth, no obvious disability, no known chronic disease (e.g., epilepsy), not hospitalized or requiring ongoing monitoring for acute malnutrition and parental consent. We conducted a house-to-house survey around each clinic and all children aged 5–23 months, living within a thirty-minute walking distance from the clinic, were screened for inclusion. We limited the sample to mothers and children living within a 30-min walk from the clinic based on prior piloting that demonstrated poor attendance among mothers living farther away (16). Children were weighed using standard methods and those with weights for age ≤ −1.5 SD of WHO standards (17) and meeting all other inclusion criteria were invited to participate in the study. We initially aimed to recruit children with a WAZ < −2.0 SD but fewer children met the criteria than anticipated. We recruited up to twenty-four children in each clinic. In clinics with more than twenty-four eligible children, a simple random sample of twenty-four children was selected. Written informed consent of mothers was collected at enrollment. Ethical approval was given by the institutional review board of the International Centre for Diarrhoeal Diseases Research, Bangladesh (icddr,b).

### Randomization

The forty clinics were stratified by subdistrict and then randomly assigned 1:1 to intervention or control by an independent statistician, using a computer-generated randomization sequence. All clinics and mother/child dyads were recruited prior to randomization. Baseline measurements were conducted after randomization. Data collectors were masked to group allocation at baseline and endline.

### Intervention

Mothers and children attending clinics allocated to the intervention group were invited to fortnightly parenting sessions for one year, held inside the clinic. The parenting sessions were facilitated by the clinic health workers. Each community clinic has three health staff: a Community Health Care Provider (CHCP) who works full time in the clinic and a Health Assistant (HA) and a Family Welfare Assistant (FWA) who work half-time in the clinic and half-time in the community. CH and HAs have masters’ degrees and most FWAs have completed high school. To promote co-ordination and cooperation, all three cadres of health staff conducted parenting sessions: CHCPs conducted 1–2 sessions per week, while HAs and FWAs who spend fewer days in the clinic conducted one session per week. Where necessary the CHCPs gave support to the FWAs. There was an average of four mother/child dyads in each group, with group size constrained by the available space within the clinic. The Group Reach-Up and Learn curriculum was used in the parenting sessions. This curriculum was adapted from the Jamaican Reach-Up home visiting program (16). The health workers were trained and supervised by the research team. See Box 1 for further details of the intervention. Mothers and children in control clinics were not invited to parenting sessions, but they used the clinic as usual for health care. We recorded attendance at parenting sessions.

### Measurements

Outcome measurements included child development, behavior, and nutritional status and mothers’ parenting knowledge and depressive symptoms, and stimulation in the home. All outcomes were measured at baseline (from September to December 2015) and after one year of intervention (from October to December 2016) and have been used previously in Bangladesh (9, 11, 18, 19). Children were tested in the presence of the mother either in a private room at the community clinic or an alternative location in the community.

#### Primary Outcomes

The primary outcomes were child development, behavior, and nutritional status. Children’s development was measured using the Bayley Scales of Infant and Toddler Development (20). We used three composite scores: (1) cognition, (2) language (combined score of the expressive and receptive language scales), and (3) motor (combined score of the fine and gross motor scales). Child behavior was rated during the test using four Wolke’s behavior rating scales: approach to examiner, emotional tone, cooperativeness, and vocalizations (21). Approach was rated during the first 10 min of the test; the remaining three scales were based on the child’s behavior throughout the test. Behaviors were rated on an 8-point scale with higher scores representing more of the characteristic. Child weight and length/height were measured by the testers after the Bayley test using WHO standard methods (22). The z scores of weight-for-age, weight-for-height, and height-for-age were calculated using WHO anthroplus (17). Children were tested at baseline and endline by one of eight testers. All testers had a Masters’ degree in Psychology or a related field. Testers received one month training and they were masked to group allocation.

#### Secondary Outcomes

The secondary outcomes were mothers’ parenting knowledge, stimulation provided in the home and mothers’ depressive symptoms. Parenting knowledge was measured using a specially designed instrument consisting of 20 questions. Stimulation in the home was measured using an extended version of the Family Care Indicators (FCI) (23). The FCI consisted of 24 questions including questions on the availability of play materials and the extent to which the mother and other adults in the home engaged the child in play activities. The FCI has been previously validated in Bangladesh and the items used in this study (variety of play materials and play activities) were shown to be highly correlated with the HOME (*r* = 0.72 and *r* = 0.73) and correlated with children’s receptive and expressive language (*r* = 0.37 to *r* = 0.48) and Bayley scores (*r* = 0.19 to *r* = 0.29) (19). Maternal depressive symptoms were measured using six questions that are included in the FCI, taken from the Center for Epidemiological Studies Depression Scale (24). All interviews with mothers were interviewer-administered and conducted after child measurements were completed.

**Description of the Group Reach-Up and Learn Parenting Intervention.**

**Content:** The Group Reach-Up and Learn curriculum focusses on: (1) improving mothers’ knowledge of parenting practices, promoting responsive parenting and stimulation in the home, and increasing mothers’ self-confidence in parenting, and (2) increasing children’s cognitive, language, motor, and behavioral development. Mothers are encouraged to engage in responsive, playful interactions with their child using low cost play materials, books and materials in the home, and in everyday caregiving routines. Activities for children under 24 months are based on the constructs of object permanence, means-end causation, vocal and physical imitation, and exploration of objects. Activities for children 24 months and older aim to teach concepts including size, quantity, color, shape, position, same/different, and classification. Activities to promote attention, persistence and problem-solving (e.g., puzzles) are also included.

**Materials:** Intervention materials for facilitators included a curriculum manual with twelve parenting sessions, to be repeated every 6 months. The curriculum is suitable for children aged 6–36 months with activities divided into four age bands (6–11, 12–18, 19–30, and 31–36 months). We also prepared summary cards for each of the 12 sessions for facilitators to use during the session (so that they didn’t have to manipulate the larger manual). Intervention materials for parents and children included picture books, play materials made from recycled materials (e.g., shakers, stacking toys, push-a-long toys, nesting toys), wooden blocks, soft toys (e.g., doll, ball, bean bag), puzzle boards, matching games, and crayon and paper. Mothers were given at least one toy and a book at each session and these were swapped for a different toy and book at the next session. Mothers also received a recipe card with examples of nutritious recipes using readily available, low-cost food.

**Procedure:** Mothers attended with their child in groups of 4–5 mother-child dyads. Each session included the following activities: (1) feedback from the previous session, (2) a song, (3) demonstration and practice of a toy, book and language activity, (4) a nutritional message, and (5) review and reminder of home activities. Facilitators demonstrated each toy, book and language activity with mothers and children grouped by age range (i.e., 6–11 months, 12–18 months, 19–30 months, 31–36 months), and supported mothers as they practiced the activities with their child. Mothers were also encouraged to share ideas for other activities and songs and to continue with the activities at home. There was a strong focus on promoting mothers’ self-efficacy and enjoyment of parenting and encouraging mothers to support and praise each other.

**Who provided:** Fifty-six frontline government health workers [20 Community Health Care Providers (CHCPs), 18 Family Welfare Assistants (FWAs) and 18 Health Assistants (HAs)] working in community clinics were trained to conduct the parenting sessions. Twelve (60%) CHCPs, nine (50%) HAs, and all eighteen FWAs were female. Health workers attended 10 days of initial training in batches of 12–15 participants and a 1-day refresher training every 3 months. We gave the health workers a stipend for attending training using the established government rates; all health workers attended the full complement of training. The training was practical and participatory and involved demonstration and simulated practice of all activities followed by a practice session with mothers and young children. Trainers were child development specialists with prior experience implementing the Reach-Up and Learn curriculum in Bangladesh. The health workers were supervised by one of five supervisors hired by the research team. Supervisors had Masters degrees in Psychology or Social Science and received 20 days of initial training from the research team. Each supervisor was responsible for four community clinics and 10–12 health workers. Supervisors visited each health worker twice a month and observed a parenting session using a checklist to monitor quality of implementation. Supervisors provided support where necessary throughout the session and at the end of the sessions, a discussion was held with the individual health worker using the checklist as a guide.

**Where:** Parenting sessions were delivered inside the community clinics. The community clinics in the study area are small buildings with no outside waiting area (see Supplementary Figure 1). It is not possible to conduct sessions in the open air near the community clinics. It hot for almost 7–8 months per year, stormy and rainy for 2–3 months and too cold for the remaining months. We had initially planned to include 6–8 children per session, but our pilot showed that there was insufficient space. Health Assistants and Family Welfare Assistants split their working time between work in the clinic and work in the community. The parenting sessions were integrated into their work in the clinic as it was easier for them to fit the sessions into their existing duties on clinic days and to keep contact with clinic activities if necessary.

**When and how much:** Each mother-child dyad was invited to participate in fortnightly parenting sessions over the period of one year (a total of 25 sessions). Session duration was 40–50 min. Mothers were given a calendar and the next session date was marked on the calendar at the end of every session. In addition, health workers communicated with the mothers via mobile phone prior to each session to remind them to attend. Supervisors assisted the health workers in making these calls when necessary. Before the start of the intervention, community motivational meetings were held in each area to encourage participation, and every 4 months, a refresher meeting was held in each village for all participating mothers and other family members to sustain engagement in the program.

**Tailoring/Modifications:** The curriculum was adapted from a home-visiting curriculum that had been used previously in Bangladesh (now called Reach-Up and Learn). We reduced the number of play materials, designed activities that were suitable for children over a wider age range rather than the age-specific activities in the original curriculum, provided guidelines and activities to promote interaction between mothers, and made the language activities more practical and concrete. The final curriculum manual was mostly implemented in a standard way by all health workers.

#### Quality Control of Measurements

Before the study assessments began, interobserver reliabilities were measured between each tester and the trainer on 8–16 tests per tester. Inter-observer reliabilities were acceptable for all measures: intraclass correlation coefficients (ICC) > 0.98 on Bayley composite scores, range of ICC = 0.62–1.00 on behavior ratings, and ICC > 0.95 on anthropometric measures. Interobserver reliabilities were conducted on approximately 10% of all Bayley tests during the study and reliabilities were ICC > 0.95 for all Bayley composite scores and ICC = 0.67–0.99 for behavior ratings.

All maternal questionnaires had good internal consistency at baseline (Cronbach’s α mean 0.82, range: 0.68–0.89) and endline (Cronbach’s α mean 0.84, range: 0.79–0.88; webtable 1). The Bayley Scales scores at baseline and endline were significantly correlated with height-for-age (*r* = 0.18–0.30), weight-for-age (*r* = 0.21–0.28) and with maternal education (*r* = 0.10–0.19) and paternal education (*r* = 0.12–0.24), indicating good discriminant validity (webtable 2).

### Statistical Analysis

The primary outcomes of the study were child development (3 scores: cognitive, language and motor development), child behavior (4 scores: approach, emotional tone, cooperativeness, vocalizations) and child nutritional status (3 scores: weight-for-age, weight-for-height, height-for-age). To calculate the sample size, we used a significance level of 0.005 (instead of 0.05) to account for 10 primary outcomes and we assumed an intracluster correlation coefficient of 0.05 (5). With an average of 21 mother/child dyads per clinic (378 mother/child dyads), and allowing for a loss of two clinics per group, (giving 18 clinics in each group), we had 80% power to detect an effect of 0.38 SD on the primary outcomes.

All analyses were prespecified. For each outcome, we fitted a multi-level random effects model that accounted for clustering at the clinic level. We adjusted for child age and sex, the relevant baseline score and tester/interviewer. Study group was entered as a binary variable. For child development and behavior outcomes, as children were tested either in the community clinic or in an alternative location in the community, we also entered place of test and an interaction term of place of test x group as fixed effects. Data completeness was excellent (>98%) for child outcomes. At endline, we had incomplete data for maternal outcomes (91% for parenting knowledge and home stimulation, 90% for maternal depression). We used multiple imputation, assuming data was missing at random, to account for missing data. Baseline sociodemographic variables and baseline scores of all child and maternal outcomes were included in the imputation model. We generated 20 datasets and ran a full multi-level random effects model using the whole dataset and to correct for overfitting, we implemented a bootstrap (200 samples) for each imputed dataset. The final models were obtained by fitting a multi-level model with all the above factors, and estimates were combined using Rubin’s rules (25). To allow for comparability across outcomes, effect sizes were calculated by using an internal standardization of the whole sample at baseline and endline separately. We used intention-to-treat analyses for all outcomes and we controlled for multiple primary outcomes using Holm step-down procedure. All analyses were carried out using Stata version 15. In *post hoc* analyses, we examined whether parenting outcomes (home stimulation, child-rearing knowledge, mothers’ depressive symptoms) mediated the effect of the intervention on child development and behavior. Baseline and endline score for each parenting outcome were entered into the multilevel regressions on child outcomes and we used a Sobel test to assess the significance of the mediation effect. The trial registration number is NCT02208531.

## Results

**FIGURE 1:**
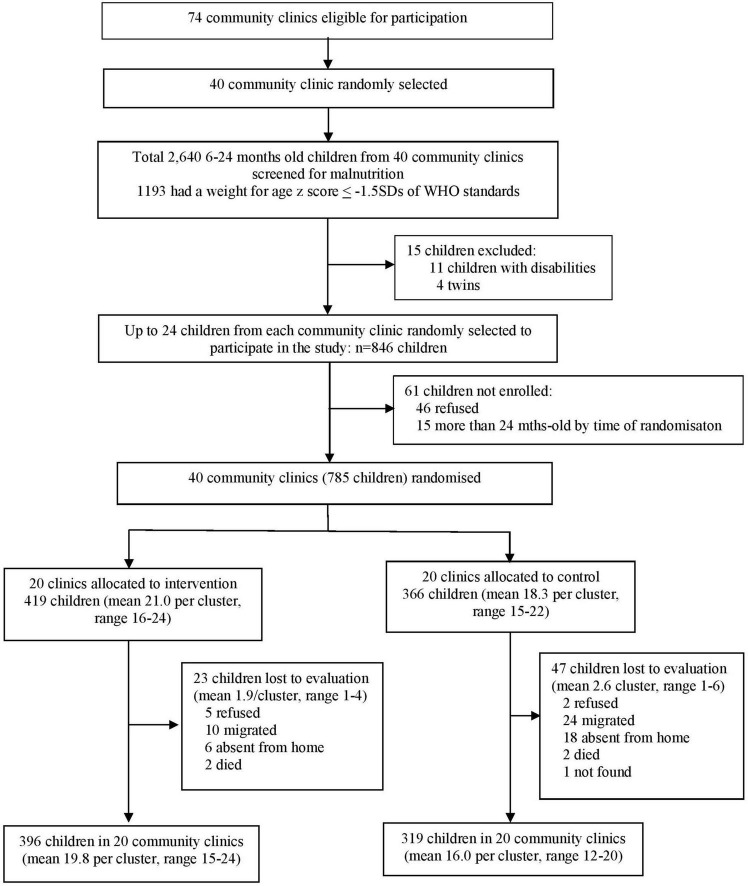
Trial profile.

We weighed 2,640 children aged 6–24 months living within a 30 min walking distance from forty community clinics. We identified 1,193 (45%) children with a weight-for-age *z*-score ≤ −1.5 SD of the WHO standard (Figure 1). We randomly selected up to 24 children from each clinic who met the inclusion criteria for the study to give a total of 846 children. Forty-six mothers (5.4%) refused to participate in the study and a further 15 children (1.8%) were more than 24-months-old by the time of randomization (due to a delay in official procedures), leaving a total of 785 children in forty clinics. Clinics were then randomly assigned to the intervention (20 clinics, 419 children) or control group (20 clinics, 366 children). All clinics were retained in the study. 70 children (8.9%) were lost at endline [23 (5.5%) intervention, 47 (12.8%) control]. Reasons for loss are shown in Figure 1. The only differences between children lost and those retained (webtable 3) were in nutritional status: children tested had lower height-for-age [Mean (SD): tested = −2.44 (1.12) vs lost = −2.03 (1.67), *p* = 0.004] and higher weight-for-height [Mean (SD) tested = −1.34 (0.10) vs lost = −1.65 (0.99), *p* = 0.02] than those lost. Groups were reasonably well-balanced at baseline with the only significant differences being higher scores for the control group on approach (*p* < 0.001), emotional tone (*p* = 0.003), and cooperation (*p* = 0.005; Table 1).

**TABLE 1:** Child and family characteristics and child and maternal outcomes at baseline and endline by study group.

	Baseline		Endline	
	Intervention *n* = 419	Control *n* = 366	Intervention *n* = 396	Control *n* = 319
**Child and family characteristics**				
Child sex: *n* (%) female	206 (49.2)	178 (48.6)	–	–
Child age (months) *n* (%)	16.89 (4.82)	17.16 (5.13)	–	–
Height-for-age < −2 *z*-scores *n* (%)	264 (63.5%)	229 (62.7%)	–	–
Weight-for-height < −2 *z*-scores *n* (%)	95 (22.7%)	80 (21.9%)	–	–
Weight-for-age < −2 *z*-scores	244 (58.5%)	214 (58.5%)	–	–
Maternal education ≥ grade 5 *n* (%)	272 (64.9%)	227 (62.0%)	–	–
Mother’s BMI	20.30 (3.07)	20.21 (3.18)	–	–
Housing	8.37 (1.78)	8.21 (1.59)	–	–
Crowding index	0.29 (0.16)	0.29 (0.18)	–	–
Monthly income ≥ 6000 BDT *n* (%)	258 (61.6%)	198 (54.1%)	–	–
**Child outcomes**				
Cognitive composite score	91.17 (11.18)	91.75 (10.93)	89.32 (6.63)	83.32 (6.63)
Language composite score	85.17 (10.39)	86.20 (10.01)	90.29 (8.55)	85.48 (7.61)
Motor composite score	90.67 (11.32)	90.83 (10.58)	93.29 (9.56)	88.76 (8.31)
Approach	5.66 (0.92)	5.87 (0.84)	5.58 (0.91)	5.19 (0.87)
Positive emotional tone	5.26 (0.79)	5.43 (0.82)	5.50 (0.80)	5.28 (0.74)
Cooperativeness	5.14 (0.89)	5.32 (0.92)	5.46 (0.84)	5.18 (0.78)
Vocalization	3.72 (1.64)	3.88 (1.70)	4.74 (1.35)	4.34 (1.29)
Height for age *z*-score	−2.43 (1.15)	−2.38 (1.12)	−2.57 (0.94)	−2.45 (1.00)
Weight for age *z*-score	−2.24 (0.85)	−2.23 (0.81)	−2.21 (0.77)	−2.26 (0.81)
Weight for height *z*-score	−1.36 (1.02)	−1.37 (0.94)	−1.12 (0.90)	−1.29 (0.91)
**Parenting and maternal depression**				
Knowledge on child rearing practices	22.55 (4.98)	22.75 (5.55)	31.22 (4.49)	23.24 (5.06)
Home stimulation	20.75 (8.12)	21.26 (7.08)	20.30 (6.36)	14.88 (6.44)
Maternal depression	8.23 (8.44)	8.17 (7.91)	6.95 (7.36)	8.22 (7.63)

*Values are mean (SD) unless otherwise stated. Housing index is the sum of ratings the quality of wall, roof and floor condition, and the presence of electricity. Child cognition, language and motor scores were measured using the Bayley Scales of Infant and Toddler Scale-version III. Response to examiner, emotional tone, cooperativeness, and vocalization were rated during the test using the Wolke’s behavior rating scales (8-point scale: 1 = low, 8 = high). Knowledge of child rearing practices was measured with a structured questionnaire used in previous studies (20 questions, potential range of scores: 0–60). Maternal depressive symptoms were measured using a shortened Center for Epidemiological Studies Depression Questionnaire (CES-D) scale (6 questions, potential range of scores: 0–42), home stimulation was assessed using Family Care Indicators (24 questions, potential range of scores: 0–24). For maternal outcomes at baseline: parenting knowledge and maternal depression: n = 417 intervention, 365 control; stimulation in the home: n = 361 intervention, n = 305 control. At endline: parenting knowledge: n = 396 intervention, n = 318 control; parenting practices: n = 393 intervention, n = 322 control; maternal depression: n = 391 intervention, n = 312 control.*

Mothers in intervention clinics attended a mean of 22.2 (SD = 5.9) parenting sessions. 215 mothers (51.3%) attended all twenty-five sessions; only 11 mothers (2.6%) attended zero sessions. Out of 56 health workers trained to conduct parenting sessions, 47 (84%) conducted all sessions. Two (3.5%) refused (both HAs) and their sessions were conducted by the CHCP at their respective CC. Seven health workers (12.5%) missed one or more sessions due to sickness, leave, or competing duties.

### Primary Outcomes

We found significant benefits of intervention to children’s score on the Bayley Scales across all developmental domains: cognitive [effect size (ES) = 0.85, 95% confidence interval (CI): 0.59, 1.11], language (ES = 0.69 95% CI: 0.43, 0.94), and motor (ES = 0.52, 95% CI: 0.31, 0.73; Table 2). We also found significant benefits of intervention for child behavior during the test, including approach (ES = 0.53, 95% CI: 0.35, 0.71), positive emotional tone (ES = 0.36, 95% CI: 0.14, 0.58), cooperativeness (ES = 0.43, 95% CI: 0.20, 0.66), and vocalizations (ES = 0.40, 95% CI: 0.26, 0.55). Children’s anthropometric measurements were not different between the groups (Table 2).

**TABLE 2:** Effect of intervention on primary and secondary outcomes at endline.

	Regression coefficient B (95% CI)	ICC	Effect size (95% CI)	*P*-value
**Primary outcomes (all child outcomes)**				
Cognitive composite score	6.17 (4.29, 8.06)	0.07	0.85 (0.59, 1.11)	0.001
Language composite score	5.81 (3.69, 7.94)	0.10	0.69 (0.43, 0.94)	0.001
Motor composite score	4.87 (2.91, 6.82)	0.03	0.52 (0.31, 0.73)	0.001
Approach	0.48 (0.32, 0.65)	0.01	0.53 (0.35, 0.71)	0.001
Positive emotional tone	0.29 (0.11, 0.46)	0.02	0.36 (0.14, 0.58)	0.001
Cooperativeness	0.35 (0.17, 0.54)	0.04	0.43 (0.20, 0.66)	0.001
Vocalization	0.52 (0.33, 0.71)	0.03	0.40 (0.26, 0.55)	0.001
Height for age *z*-score	–	0.06	−0.16 (−0.31, −0.01)	0.06
Weight for age *z*-score	–	0.05	0.04 (−0.08, 0.15)	0.52
Weight for height *z*-score	–	0.05	0.20 (0.04, 0.35)	0.06
**Secondary outcomes (parenting outcomes)**				
Child-rearing knowledge	7.87 (7.00, 8.73)	–	1.27 (1.13, 1.41)	<0.001
Home stimulation	5.35 (4.14, 6.56)	–	0.77 (0.60, 0.94)	<0.001
Maternal depression	−1.39 (−2.54, −0.23)	–	−0.18 (−0.34, −0.03)	0.02

*ICC, intracluster correlation coefficient. 1 = intervention, 0 = control. Analyses were adjusted for child age and sex, tester/interviewer, baseline score as fixed effects and community clinic as a random effect. Analyses for child development and behavior outcomes also included place of test, and a place of test x group interaction term as fixed effects. p values for all 10 primary outcomes have been corrected for with Holm’s stepdown procedure.*

### Secondary Outcomes

Mothers in intervention clinics had significantly better parenting knowledge (ES = 1.27, 95% CI: 1.13, 1.41) and fewer depressive symptoms (ES = −0.18, 95% CI: −0.34, −0.03), than mothers in control clinics (Table 2). We also found significant benefits of intervention for home stimulation as measured by the FCI (ES = 0.77, 95% CI: 0.60, 0.94).

### *Post hoc* Analyses

In mediation analyses, home stimulation and mothers’ child-rearing knowledge significantly mediated the effect of intervention on child development (Bayley Scales) and behavior (Wolke behavior ratings; **webtables 4** and **5**), whereas maternal depressive symptoms was not a significant mediator of child outcomes (**webtable 6**).

## Discussion

We integrated an ECD, group-based parenting program into government primary health care clinics in rural Bangladesh, with parenting sessions conducted by existing health workers as part of their usual duties. Many of the enrolled children were moderately malnourished and at high risk for poor development. We found significant benefits to child cognitive, language and motor development, and to child behavior with children in the intervention group rated as happier, more sociable, more cooperative and more vocal during the developmental test session. There were no benefits to children’s nutritional status. Mothers in the intervention clinics reported higher levels of stimulation in the home, better parenting knowledge and fewer depressive symptoms than mothers from control clinics.

The moderate to large benefits to child development and behavior found in this study are considerably larger than those found in Bangladeshi studies that used a similar curriculum, but delivered by local women, in individual home or clinic sessions (9–12). The benefits are also larger, (approximately double), than those reported in a recent meta-analysis of childhood parenting interventions that reported mean effect sizes and 95% CI of 0.41 (0.29, 0.53), 0.35 (0.21, 0.48), 0.26 (0.16, 0.36) for child cognitive, language and motor development, respectively, from studies in LMIC (2). The behavior ratings in the present study are not strictly comparable to the socio-emotional development measures but they also tended to have higher impacts.

In the previous Bangladeshi trial (pair study) using a similar delivery model but where pairs of mothers and children participated in the sessions, treatment effects were even larger on child development (ranging from 1.1 to 1.3 SD) and child behavior (ranging from 0.7 to 1.1 SD) (13). The moderate to large treatment effects found in the present trial partially replicates those findings but are somewhat smaller. The difference in treatment effects may reflect differences in delivery of the intervention. It is likely that groups of four are more challenging to handle than groups of two. Also in the pair study, play activities were more closely targeted to each individual child’s developmental level and staff used a more detailed curriculum. To make the program more feasible at scale, the groups of four used fewer play materials and play activities were adapted for use across a wider age range. Another possibility is that children in the pair study were slightly more disadvantaged with more children being moderately undernourished and the parents being less educated compared with the present study. There is some evidence that interventions benefit disadvantaged children more than less disadvantaged ones (26).

The larger benefits in both the present and the pair studies compared with individual intervention sessions may be due to several factors. The parenting sessions were delivered by government health workers who were better educated than paraprofessional facilitators and are well respected in the community, which may enhance credibility. Group sessions provide mothers with the opportunity to engage in peer learning and gain social support and may reduce feelings of isolation and promote group norms that support responsive and playful parenting (27–29). We also placed strong emphasis on making the sessions fun and interactive for mothers and children and group sessions may be more enjoyable than individual sessions in this context, thus leading to higher participant engagement and motivation (30–32). Group-based ECD parenting interventions have been shown to be effective in other contexts, including when integrated into existing services and delivered by existing staff (7, 33, 34), and when implemented by community volunteers trained specifically to deliver the program (35–37). In addition, there is some evidence that group-based parenting interventions are more cost-effective than individual home-visiting and mixed group and home-visiting delivery models, leading to increased scalability (38, 39). Benefits to child development and parenting outcomes are more likely to be sustained when the immediate impacts are larger, and the moderate-to-large effect sizes reported in this and the pair study are encouraging (40). We are currently planning a follow-up study to examine whether benefits are sustained.

The treatment effect on child cognitive, language and motor development and behavior during the test were mediated by increases in mothers’ parenting knowledge and stimulation in the home. This is expected as the intervention aims to promote child development by supporting mothers in responsive and playful parenting practices (41). Although we found significant reductions in maternal depressive symptoms, this reduction did not mediate the impact on child outcomes. Maternal depression is less commonly measured in evaluations of ECD programmes. but the studies available indicate that these programs have potential to benefit maternal mental health, further strengthening their value (42). However, benefits have not been found consistently (2).

There were no benefits from the parenting intervention on children’s nutritional status. The parenting sessions included a nutrition education component and mothers were provided with a recipe booklet with low-cost, nutritious recipes suitable for undernourished children. Over 62% of children were moderately stunted and 58% moderately underweight at baseline. For gains to children’s nutritional status, food supplementation is likely to be necessary and is more effective if begun in the first year before undernutrition develops (43, 44).

The study has several strengths including the use of a cluster-randomized study design leading to well-balanced groups, prespecified analyses, masked assessors, intention-to-treat analyses, adjustment for multiple outcomes, the use of direct assessments of child development and observational measures of child behavior. The outcome measures had good psychometric properties and although the Bayley scales are not standardized for Bangladesh, they have good concurrent and predictive validity and scores correlate with child nutritional status and maternal and paternal education in a logical way in this population (8, 13). The study also had some limitations. Stimulation in the home was assessed through self-report and hence may be subject to bias. Although assessors were masked to intervention group, it is possible that some mothers may have mentioned the intervention during endline data collection.

The intervention was implemented in government community clinics by government health workers, used low-cost play materials and activities, and was acceptable to mothers and health staff as shown by the high compliance and engagement in the intervention. These factors make it suitable for wider dissemination within Bangladesh. The community clinics that participated in this study are similar to those in other areas of rural Bangladesh and hence the results should generalize to clinics across the country. However, there are some limitations to consider as the program is scaled-up. Firstly, the research team trained and supervised the health staff which helped ensure high quality intervention implementation. In future, it will be important to test if implementation quality is maintained when government health supervisors provide the training and supervision or if it is necessary to hire new supervisors for child development. Secondly, health staff participated in 10 days initial training and quarterly 1-day refresher trainings. This is lower than reported in many other group-based ECD programmes (33–35, 38), although longer than may be readily available in many government programs. High quality training and ongoing supervision is a key requirement for quality implementation and we need to advocate for sufficient training as ECD programmes are scaled-up. Thirdly, provision of play materials is a core component of Reach-Up and Learn and is essential to maintain effectiveness of the intervention as suggested by a Madagascan study, which used the Reach Up curriculum without leaving toys with the mothers, and found no impact on child development (45). In this and previous studies, toys have been provided by the research team. Others have implemented effective program that require parents to provide home-available playthings for their children (4, 7, 33, 46). Hence, we can explore to what extent the play materials can be made by the mothers or communities. Another limitation is the relatively small number of children who can be reached through this model. The existing 13,000 clinics could reach approximately 416,000 children a year. Therefore, this approach is targeted to the highest risk children only and there remains a need to explore ways of increasing coverage, including increasing the group size where practical. In this study and the pair study, we targeted undernourished children because undernutrition is an important risk factor for poor child development. Alternative strategies would be required for high-risk children living farther from the clinic. For example, ECD content could be integrated into the home visits and community health sessions conducted by FWAs and HAs as has been reported in other studies in Bangladesh (37, 47).

In conclusion, our results suggest that integrating an ECD parenting intervention into government primary health care services in rural Bangladesh was feasible and effective for groups of four mothers and children making the program most suitable for targeting high-risk children. The intervention has the potential to be scaled up to other areas thus increasing the coverage of ECD programming for disadvantaged children.

## Data Availability Statement

The raw data supporting the conclusions of this article will be made available by the authors, without undue reservation.

## Ethics Statement

The studies involving human participants were reviewed and approved by Institutional Review Board of International Centre for Diarrhoeal Disease Research, Bangladesh. Written informed consent to participate in this study was provided by the participants’ legal guardian/next of kin.

## Author Contributions

JH, SG-M, HB-H, and SFM contributed to the conceptualisation of the study and funding acquisition. SFM and JH contributed to the project administration. SFM, JH, MH, SS, FT, HB-H, and SG-M contributed to the investigation. DR was responsible for data analysis. SFM and HB-H were responsible for writing the original draft. All authors reviewed and edited the manuscript.

## Conflict of Interest

The authors declare that the research was conducted in the absence of any commercial or financial relationships that could be construed as a potential conflict of interest.

## Publisher’s Note

All claims expressed in this article are solely those of the authors and do not necessarily represent those of their affiliated organizations, or those of the publisher, the editors and the reviewers. Any product that may be evaluated in this article, or claim that may be made by its manufacturer, is not guaranteed or endorsed by the publisher.
