# Supplementary material for: Integrating a Group-Based, Early Childhood Parenting Intervention Into Primary Health Care Services in Rural Bangladesh: A Cluster-Randomized Controlled Trial
Source: Front Pediatr. 2022 Jun 10;10:886542. doi: 10.3389/fped.2022.886542 (PMC9245711; doi:10.3389/fped.2022.886542)

Supplementary Material for:

Integrating a Group-Based Early Childhood Parenting Intervention Into Primary Health Care Services in Rural Bangladesh: A Cluster-Randomized Controlled Trial

Syeda Fardina Mehrin, Mohammed Imrul Hasan, Fahmida Tofail, Shamima Shiraji, Deborah Ridout, Sally Grantham-McGregor, Jena D Hamadani, Helen Baker-Henningham

Webtable 1. Internal reliablities of questionnaires at baseline and endline

	Internal Reliability (Cronbach's alpha)	
Outcome measure	Baseline	Endline
Parenting knowledge	0.68	0.79
Family Care Indicators (Home stimulation)	0.89	0.86
Maternal depressive symptoms	0.88	0.88

Webtable 2. Correlations of child nutritional status and parental education with the Bayley test scores

Bayley Scales Scores	Maternal education (years)	Fathers' education (years)	Height-for-age z score at baseline	Weight-for-age z score
Cognitive composite baseline	0.10*	0.12*	0.28**	0.24**
Language composite baseline	0.19**	0.24**	0.28**	0.22**
Motor composite baseline	0.16**	0.17**	0.30**	0.25**
Cognitive composite endline	0.17**	0.13**	0.18**	0.21**
Language composite endline	0.12**	0.23**	0.22**	0.28**
Motor composite endline	0.16**	0.17**	0.24**	0.23**

*p<0.01, **p<0.001

Webtable 3. Child, family and maternal characteristics by loss at endline

	**Tested (n=715)**	**Lost (n=70)**	**P value**
**Child characteristics**			
Child age	17.02 (4.99)	16.94 (4.70)	0.90
Child sex: female *n (%)*	347 (48.5%)	37 (52.9%)	0.49
Cognition	91.34 (11.10)	92.50 (10.66)	0.40
Language	85.56 (10.26)	86.57 (9.92)	0.43
Motor	90.51 (11.01)	93.14 (10.38)	0.06
Approach	5.76 (0.88)	5.71 (0.95)	0.69
Positive emotional tone	5.34 (0.81)	5.26 (0.81)	0.40
Cooperativeness	5.23 (0.92)	5.17 (0.80)	0.61
Vocalization	3.79 (1.70)	3.77 (1.45)	0.92
WAZ	-2.24 (0.84)	-2.22 (0.77)	0.88
HAZ	-2.44 (1.12)	-2.03 (1.67)	0.004
WHZ	-1.34 (0.10)	-1.65 (0.99)	0.02
**Family and maternal characteristics**			
Housing index	8.28 (1.67)	8.46 (1.95)	0.49
Crowding	0.29 (0.17)	0.30 (0.19)	0.80
Income $\geq$ 6,000 BDT *n (%)*	349 (57.7%)	38 (62.3%)	0.49
Mother's education $\geq$ grade 5 *n (%)*	386 (63.8%)	37 (60.7%)	0.63
Maternal depression	8.06 (8.11)	9.66 (8.93)	0.12
Knowledge of child rearing practices	22.67 (5.30)	22.37 (4.66)	0.65
Home stimulation	20.94 (7.59)	21.38 (8.40)	0.67

Values are mean (SD) unless otherwise stated.

Webtable 4. Mediation analyses with stimulation in the home (Family Care Indicators)

	**BAYLEY SCALES COMPOSITE SCORES**			**WOLKE BEHAVIOUR RATINGS**			
	**Cognitive composite**	**Language Composite**	**Motor Composite**	**Approach**	**Emotional Tone**	**Cooperativeness**	**Vocalisations**
Intervention	5.86	3.54	3.69	0.34	0.17	0.19	0.27
B (95% CI)	(4.72, 7.00)	(2.19, 4.90)	(2.13, 5.26)	(0.18, 0.49)	0.03, 0.31)	(0.05, 0.33)	(0.06, 0.49)
P-value	P<0.001	P<0.001	P<0.001	P<0.001	P=0.02	P=0.01	P=0.18
Family Care Indicators	0.10	0.28	0.20	0.02	0.01	0.02	0.02
B (95% CI)	(0.02, 0.19)	(0.18, 0.37)	(0.08, 0.31)	(0.01, 0.03)	(0.00, 0.02)	(0.01, 0.03)	(0.00, 0.03)
P-value	P=0.02	P<0.001	P<0.001	P=0.003	P<0.001	P<0.001	P=0.03
Sobel-test statistic	2.43	4.89	3.16	3.16	1.96	3.72	2.43
P-value	P=0.02	P<0.001	P=0.002	P=0.002	p=0.05	P<0.001	P=0.02

1=intervention, 0=control. The dependent variables were child development measured using the Bayley Scales (cognitive, language and motor composite scores) and child behaviour using Wolke behaviour ratings (approach, emotional tone, cooperativeness, vocalisations). Independent variables were child age and sex, tester, baseline score, place of test, a place of test x group interaction term, baseline and endline score for the Family Care Indicators and study group. All analyses were multi-level models controlling for community clinic as a random effect.

Webtable 5. Mediation analyses with mothers' child rearing knowledge

	**BAYLEY SCALES COMPOSITE SCORES**			**WOLKE BEHAVIOUR RATINGS**			
	**Cognitive composite**	**Language Composite**	**Motor Composite**	**Approach**	**Emotional Tone**	**Cooperativeness**	**Vocalisations**
Intervention	3.71	1.68	1.67	0.18	-0.11	0.05	0.06
B (95% CI)	(2.22, 5.20)	(-0.22, 3.58)	(-0.29, 3.63)	(-0.05, 0.41)	(-0.30, 0.08)	(-0.16, 0.26)	(-0.18, 0.30)
P-value	P<0.001	P=0.08	P=0.10	P=0.13)	P=0.27	P=0.65	P=0.61
Child rearing knowledge	0.30	0.41	0.37	0.04	0.04	0.03	0.04
B (95% CI)	(0.20, 0.40)	(0.30, 0.52)	(0.24, 0.50)	(0.02, 0.05)	(0.03, 0.06)	(0.02, 0.05)	(0.02, 0.06)
P-value	P<0.001	P<0.001	P<0.001	P<0.001	P<0.001	P<0.001	P<0.001
Sobel-test statistic	5.53	6.48	5.38	4.95	6.93	5.55	4.28
P-value	P<0.001	P<0.001	P<0.001	P<0.001	P<0.001	P<0.001	P<0.001

1=intervention, 0=control. The dependent variables were child development measured using the Bayley Scales (cognitive, language and motor composite scores) and child behaviour using Wolke behaviour ratings (approach, emotional tone, cooperativeness, vocalisations). Independent variables were child age and sex, tester, baseline score, place of test, a place of test x group interaction term, baseline and endline score for the mothers' child rearing knowledge and study group. All analyses were multi-level models controlling for community clinic as a random effect.

Webtable 6. Mediation analyses with mothers' depressive symptoms

	BAYLEY SCALES COMPOSITE SCORES			WOLKE BEHAVIOUR RATINGS			
	**Cognitive composite**	**Language Composite**	**Motor Composite**	**Approach**	**Emotional Tone**	**Cooperativeness**	**Vocalisations**
Intervention	5.88	4.59	4.43	0.43	0.23	0.30	0.37
B (95% CI)	(4.51, 7.25)	(2.75, 6.43)	(2.64, 6.22)	(0.22, 0.64)	(0.06, 0.40)	(0.12, 0.48)	(0.18, 0.56)
P-value	P<0.001	P<0.001	P<0.001	P<0.001	P=0.01	P=0.001	P<0.001
Mothers' depressive symptoms	-0.06	-0.09	-0.11	-0.01	-0.01	-0.01	-0.02
B (95% CI)	(-0.13, 0.01)	(-0.06, -0.16)	(-0.19, -0.03)	(-0.02, -0.00)	(-0.12, 0.00)	(-0.02, -0.00))	(-0.03, -0.01)
P-value	P=0.10	P=0.01	P=0.01	P=0.001	P=0.08	P=0.001	P=0.005
Sobel-test statistic	1.25	1.49	1.49	1.68	1.29	1.68	1.61
P-value	P=0.21	P=0.14	P=0.14	P=0.09	p=0.20	P=0.09	P=0.11

1=intervention, 0=control. The dependent variables were child development measured using the Bayley Scales (cognitive, language and motor composite scores) and child behaviour using Wolke behaviour ratings (approach, emotional tone, cooperativeness, vocalisations). Independent variables were child age and sex, tester, baseline score, place of test, a place of test x group interaction term, baseline and endline score for the mothers' depressive symptoms and study group. All analyses were multi-level models controlling for community clinic as a random effect.

Supplementary Figure 1. A Community Clinic

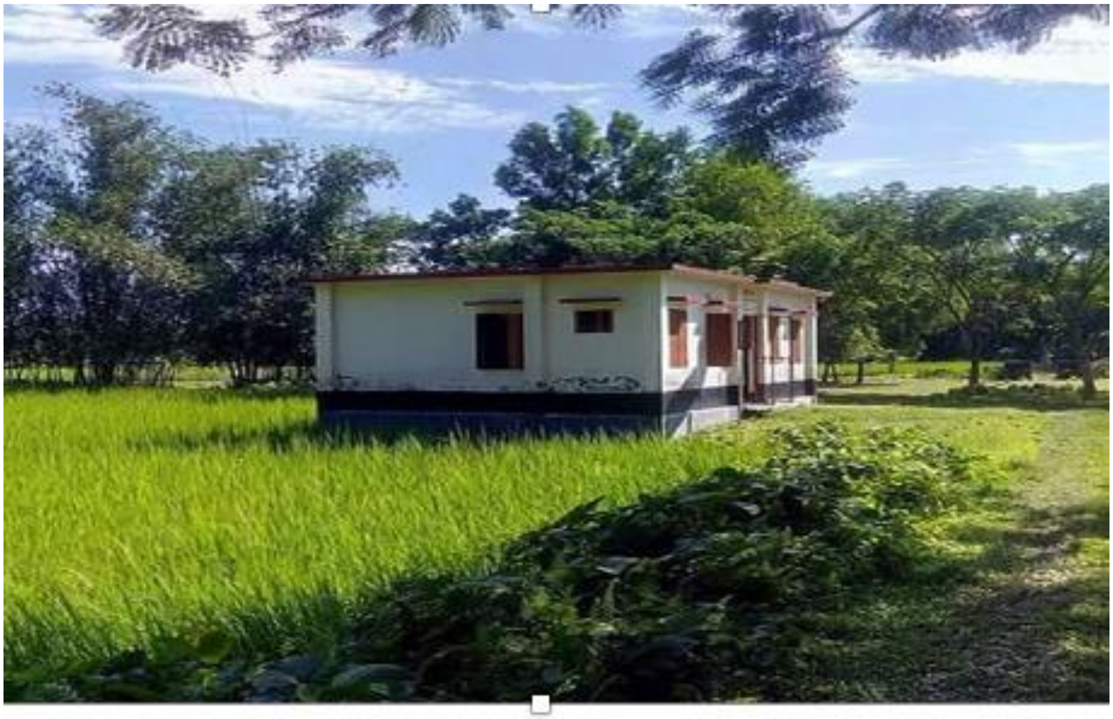

Supplement: Supplementary file 1 [file Data_Sheet_1.pdf]
